# Insurance Committees

**Published:** 1912-12-07

**Authors:** 


### NATIONAL INSURANCE ACT DEPARTMENT.

### XXXV.—Insurance Committees.

Upon the completion of the negotiations between the Chancellor of the Exchequer and the medical profession, which is now confidently expected to be reached within the course of a few days, the duties and functions of the Insurance Committees will come into prominence. So much public attention has been devoted to the formation of approved societies and to the part that they are to play in the administration of the benefits of the Act and to the importance of all insured persons becoming members of such societies, that the position of the Insurance Committees has been treated as though of relatively small importance and has excited little public comment. In point of fact, however, the duties of the Insurance Committees are almost equal in importance to those of the approved societies, and it is particularly desirable that hospital managers and others who are intimately concerned with the provision of adequate medical treatment, as well as all those of our readers who are to benefit by the Act, should have a clear exposition of the functions of the Insurance Committees and of their relation to the approved societies. It is the intention of this article to furnish such a statement.

### Insurance Committees and Medical Benefit.

In the Bill as originally presented to Parliament it was proposed to set up local Health Committees, whose principal duties were to be the administration of the sanatorium benefit and the general oversight of health conditions in the counties and county boroughs for which they were to be formed. They were also to administer the benefits of deposit contributors and to share with the approved societies the duty of administering medical benefit. It may be remembered that the last proposal met with considerable opposition from the medical profession, and in consequence an amendment of fundamental importance was introduced into the measure, which finally became incorporated into the Act, by which the administration of medical benefit was entirely excluded from the management of the approved societies and transferred to the local Health Committees. At the same time the functions of the latter were in certain other ways modified and expanded, and by one of the last amendments to receive the sanction of the House the description of "local Health Committee" was altered to "Insurance Committee," as it now stands in the Act.

### The Constitution of Insurance Committees.

The constitution, powers and duties, and sources of income of the Insurance Committees are laid down in Sections 59, 60, and 61, but in addition to these provisions there are constant references in the Act to the Insurance Committees, and in particular Sections 15 to 17, which deal with the administration of medical and sanatorium benefits, are almost entirely concerned with the Insurance Committees.

An Insurance Committee has been constituted for each county and county borough throughout the United Kingdom. They have already commenced their labours, having been appointed by regulations of the Insurance Commissioners in June last.

The number of members constituting an Insurance Committee depends upon the population of the county or county borough it represents, the minimum being forty and the maximum eighty. In each case three-fifths of the members represent the insured persons resident in the Committee area, and one-fifth are appointed by the Council of the county or county borough. The remaining fifth is made up as follows: (*a*) Two members are elected by the local medical practitioners; (*b*) either one, two, or three, according to the size of the Committee, are to be duly qualified medical practitioners appointed by the Council of the county or county borough; and (*c*) the remaining members are appointed by the Insurance Commissioners.

The representatives of the insured persons have to be chosen from the approved societies as nearly as may be in proportion to the number of members of such societies resident in the Committee area, and deposit contributors are likewise represented in proportion to the local membership in the Post Office Fund. It may here be mentioned that as there was no means of knowing the number of insured persons likely to become members of individual societies at the time the present Committees were appointed, and that it was absolutely impossible to gauge the possible membership in the Post Office Fund, the present Committees are of a somewhat tentative character, and some time must necessarily elapse before they can be made truly representative of the insured persons.

So far as the members appointed by the local Council are concerned, it is provided in the Act that two at least shall be women, and of those appointed by the Insurance Commissioners one at least must be a qualified medical practitioner and two at least shall be women.

It will be seen that the Insurance Committees are constituted in such a way as to give predominance to the representatives of the insured persons, and, although we learn that in most cases the Committees have elected one of the members appointed by the Council of the county or county borough as chairman of the Committee, it is clear that the representatives of the insured persons—that is to say, in effect, the executives of the approved societies—have the power of out-voting all other interests on the Committee should occasion demand. This is an important point, and in some degree it is responsible for the protracted negotiations between the Chancellor of the Exchequer and the medical profession, for as medical benefit is to be administered by the

Insurance Committees, the Act, as it stands, places the doctors under lay control—a condition to which the profession has substantial reasons for declining.

Administration of Sanatorium Benefit.

The activities of the Insurance Committees up to the present have been mostly confined to the settlement of procedure—election of sub-committees, appointment of officials, and making arrangements in connection with the provision of sanatorium treatment for insured persons suffering from tuberculosis. We need not deal at length with the duties of the Insurance Committees in regard to sanatorium treatment. It will suffice to point out that they are not responsible for the erection of sanatoria, but are merely concerned with making arrangements with institutions that are prepared to receive patients and which satisfy the requirements of the Insurance Commissioners, and that no insured person is entitled to sanatorium benefit under the Act unless recommended for such benefit by the local Insurance Committee. Some criticism has been levelled against the Government on the ground of the unpreparedness of the Committees to administer the sanatorium benefit, but it is no fault of the Committees that they have been unable to provide treatment for every applicant in view of the lateness of their appointment. A commencement has, however, been made, and the Chancellor referred with pride in his Aberdeen address to two cases that had been brought to his notice.

Administration of Medical Benefit.

The principal duty of the Insurance Committees has already been alluded to—namely, that of administering medical benefit. The Act lays upon each Insurance Committee the duty of obtaining a panel of doctors who are prepared to undertake the attendance and treatment of insured persons, and to arrange for such treatment in accordance with regulations made by the Commissioners. Up to the present provisional regulations only have been issued by the Commissioners, and it is admitted that they will have to be altered in certain vital particulars in order to gain acceptance by the profession, and, in consequence, although medical benefit is promised under the Act as from January 15 next, the detailed arrangements for the provision of that benefit that will have to be made by the Insurance Committees have had to be deferred.

As between the Insurance Committees and the insured persons satisfactory progress has been made with the administrative detail. Each approved society has been required to furnish to each Insurance Committee, in whose area any of its members reside, an account of its local membership. This has been done by writing on a separate card for each member, known as an index slip, the full name and address of the member, with the name and number of the society and membership number therein. Similar index slips are being furnished for deposit contributors. When these returns are complete the Insurance Committee will be in possession of a full record of each insured person resident in its area for whom it is responsible in the matter of providing medical benefit, and will further be able to ascertain the number of such persons belonging to any one approved society. The names having been furnished on separate cards, the work entailed by transfer to another Committee, by removal of the insured person to another district, will be facilitated.

Insurance Committees and Approved Societies.

According to the letter of the Act (Section 15, sub-section 6) the necessary funds to enable the Insurance Committees to administer medical benefit are to be credited to them each year out of the funds credited to the approved societies in the books of the Commissioners, as obtained from the contributions of the members, and the sum so credited to an Insurance Committee is to be *agreed between the society and the Committee*, or, in default of agreement, is to be determined by the Commissioners.

The words we have placed in italics imply that the cost of medical benefit is to be a matter for negotiation between the societies and Committees, but this is not so in point of fact. There is nothing in the Act itself to limit the expenditure of an approved society in regard to the provision of medical benefit through the Insurance Committee, but the estimates prepared by the Government actuaries showed that a sum of 6s. per member per annum had been allowed out of the contributions for this benefit, and that the remainder of the contributions were required for the other benefits promised. The adequacy of this allowance of 6s. is not an actuarial matter, but as it was the sum used in the calculations, on the authority of the Chancellor of the Exchequer, it follows that no larger sum can be agreed to by an approved society without depleting the funds upon which it has to rely for the payment of sickness benefit. It is thus apparent that there is no room for negotiation, and the additional allowance of 2s. 6d. per member per annum recently offered by the Chancellor of the Exchequer will have to be provided from general taxation, quite apart from the charges on the National Revenue for grants authorised by the Act. The funds out of which sanatorium benefit are to be payable are a definite charge on the approved societies. The charge per member is 1s. 3d. a year, and the same charge applies to deposit contributors. An additional sum of 1d. per member per annum is to be paid out of moneys specially provided by Parliament.

The administrative expenses of the Committee are to be charged against the funds of the approved societies to the extent of 1d. per member per annum, with a further possible increase to 2d. per member per annum should it be found necessary to refund to the members of the Committee their travelling expenses.

The delay that has arisen through the protraction of the negotiations between the Chancellor of the Exchequer and the medical profession will have the effect of throwing an immense amount of work on the officials of the Insurance Committees—to be done in very short time—if the administrative detail is to be in any way complete by the middle of January.

